# Protein misfolding: understanding biology to classify and treat synucleinopathies

**DOI:** 10.1007/s00702-025-02889-0

**Published:** 2025-02-11

**Authors:** Tiago Fleming Outeiro, Günter Höglinger, Anthony E. Lang, Tuane C. R. G. Vieira

**Affiliations:** 1 Department of Experimental Neurodegeneration, Center for Biostructural Imaging of Neurodegeneration, University Medical Center Göttingen, Göttingen, Germany; 2 Translational and Clinical Research Institute, Faculty of Medical Sciences, Newcastle University, Framlington Place, Newcastle Upon Tyne, NE2 4HH UK; 3 German Center for Neurodegenerative Diseases (DZNE), Von-Siebold-Str. 3a, 37075 Göttingen, Germany; 4 Max Planck Institute for Multidisciplinary Sciences, Göttingen, Germany; 5 Department of Neurology, LMU University Hospital, Ludwig-Maximilians-Universität (LMU) München, Munich, Germany; 6 German Center for Neurodegenerative Diseases (DZNE), Munich, Germany; 7 Munich Cluster for Systems Neurology (SyNergy), Munich, Germany; 8 Edmond J. Safra Program in Parkinson’s Disease, the Rossy PSP Centre, Division of Neurology, Department of Medicine, University Health Network, University of Toronto, Toronto, Canada; 9 Institute of Medical Biochemistry Leopoldo de Meis, National Institute of Science and Technology for Structural Biology and Bioimaging, Federal University of Rio de Janeiro, Rio de Janeiro, RJ 21941-901 Brazil; 10 Department of Experimental Neurodegeneration, University Medical Center Göttingen, Waldweg 33, 37073 Göttingen, Germany

**Keywords:** Protein aggregation, Alpha-synuclein, Parkinson’s disease, Neurodegeneration, Biomarkers, Disease classification

## Abstract

Protein misfolding and aggregation is a major pathological hallmark in a variety of human conditions, including cancer, diabetes, and neurodegeneration. However, we still do not fully understand the role of protein accumulation in disease. Interestingly, recent breakthroughs in artificial intelligence (AI) are having a tremendous impact on our ability to predict three-dimensional protein structures and understand the molecular rules governing protein folding/misfolding. This progress will enable us to understand how intrinsic and extrinsic factors trigger protein misfolding, thereby changing protein function. These changes, in some cases, are related to normal biological responses and, in other cases, associated with pathological alterations, such as those found in many neurodegenerative disorders. Here, we provide a brief historical perspective of how findings in the field of prion diseases and prion biology have enabled tremendous advances that are now forming the basis for our understanding of disease processes and discuss how this knowledge is now emerging as central for our ability to classify, diagnose, and treat devastating neurodegenerative disorders such as Parkinson’s and Alzheimer’s diseases.

## Introduction

### Protein structure and function in biology and pathology

Modern biology is founded on the understanding of molecular mechanisms that govern life. Advancements in omics technologies and molecular imaging technologies, which now allow the observation of biological phenomena across various scales. These advancements allow us to study the atomic arrangement of amino acids in proteins to cellular communication and inter-organ interactions within an organism. As a result, our understanding of biological processes has evolved rapidly.

The genomic revolution has significantly enhanced our ability to identify genes, both protein-coding and non-coding genes. For protein-coding genes, it is now trivial to translate genetic information into functional information, such as protein sequences. Despite recent breakthroughs in artificial intelligence (AI), which have achieved remarkable success in predicting protein three-dimensional structures (Jumper et al. 2021) and new insights for unraveling the molecular rules governing protein folding (Chowdhury et al. 2022), a substantial gap in our understanding of the relationship between protein structure and function remains (Outeiro and Vieira 2023). Nonetheless, it is well established that protein structure is intimately tied to function and that structural alterations can lead to functional impairments, potentially disrupting biological systems in which these proteins operate. In extreme cases, such structural changes can lead to protein misfolding and aggregation, triggering cellular responses that may either confer adaptive advantages or compromise cellular viability.

Over the years, many studies have focused on unraveling the molecular mechanisms underpinning protein misfolding and aggregation, particularly in the context of pathologies characterized by the accumulation of protein aggregates. Neurodegenerative disorders, including Parkinson’s disease (PD), represent a prime example, alongside diabetes, certain types of cancer, and even certain viral infections. Recent studies revealed that the process of protein aggregation is far more complex than previously thought. Intriguingly, some forms of protein aggregation appear to play functional roles, providing adaptive flexibility to cells and to subcellular compartments under specific conditions (Hoffmann et al. 2023; Otzen and Riek 2019). This challenges the long-standing view that all protein aggregation is inherently pathological, suggesting that some proteins may acquire novel functions via aggregation.

Decades of research have significantly deepened our understanding of protein misfolding and aggregation, highlighting the central role these processes play in neurodegenerative diseases. This paper builds on this growing body of knowledge to propose new perspectives on how these processes can inform the biological classification of these complex disorders, opening approaches to designing therapeutic strategies that directly target the molecular mechanisms driving protein aggregation and associated cellular dysfunction.

### Prions: a single genome, ‘multiple’ phenotypes

The pioneering studies from Stanley Prusiner in the 1980s revolutionized our understanding of a group of devastating diseases affecting both animals and humans, collectively known as transmissible spongiform encephalopathies (TSEs). Prusiner´s work led to the formulation of the protein-only hypothesis, a paradigm-shifting idea that challenged traditional views of infectious agents by proposing that these diseases are caused not by viruses, bacteria, or other nucleic acid-based pathogens, but by a misfolded protein. To describe this novel infectious entity, Prusiner introduced the term ‘prion’ – derived from “proteinaceous infectious particle” – referring to the prion protein (PrP) as the key agent in TSEs (Prusiner 1982, 1998).

A landmark discovery in this field was the realization that prion proteins exist naturally in the host organism in a normal, non-pathogenic conformation, referred to as cellular prion protein (PrP^C^) (Basler et al. 1986). However, this protein undergoes a dramatic conformational change in TSEs into a misfolded, pathogenic form known as PrP scrapie (PrP^Sc^). This pathological form of the protein has the remarkable ability to act as a template, inducing the misfolding of normal PrP^C^ into PrP^Sc^ (Scott et al. 1989). This self-propagating cycle of protein misfolding not only explains the infectious nature of prions but their capacity to accumulate and propogate in tissues, leading to widespread neurodegeneration (Cobb and Surewicz 2009).

The ability of prions to convert the host’s normal proteins into the misfolded state also gives rise to a new phenotype in affected organisms. In prion diseases, this phenotype manifests as spongiform degeneration of brain tissue, with symptoms ranging from cognitive impairment to severe motor dysfunction, depending on the specific prion strain and host (Baiardi et al. 2019). A fascinating aspect of prions is their strain variability, where different misfolded conformations of PrP^Sc^ give rise to distinct disease phenotypes, even though the underlying amino acid sequence of PrP remains unchanged (Bruce 2003; Caughey 2003).

The prion concept was then extended to phenomena that Mendelian genetics could not explain, observed in the budding yeast *S. cerevisiae* (Patino et al. 1996; Wickner 1994). In particular, yeast cells with the same genome could exhibit different phenotypes, which was not explained by conventional epigenetic mechanisms. The phenotypes arose because the proteins Ure2p Sup35p could exist in either soluble states [*prion-*] or aggregated states [*prion+*] (Wickner 1994). These distinct biochemical states of the protein were found to confer adaptive advantages to yeast cells growing in different environments, introducing the concept of protein-based epigenetics (True et al. 2004; True and Lindquist 2000).

Despite these early advances, the precise structure of the infectious prion protein remained elusive for decades. It was only through the advent of high-resolution structural biology techniques, such as cryo-electron microscopy and advanced spectroscopy, that researchers resolved the molecular architecture of PrP^Sc^ (Kraus et al. 2021). These studies demonstrated the amyloid-like properties of PrP^Sc^, showing that its pathological form consists of parallel in-register intermolecular packed β-sheets that confer remarkable stability and resistance to degradation (Kraus et al. 2021; Manka et al. 2023). This structural discovery provided strong evidence supporting the protein-only hypothesis and highlighted the similarities between prions and other protein aggregates linked to disease.

The significance of prions extends far beyond their role in transmissible spongiform encephalopathies. Their study has opened new avenues for understanding numerous neurodegenerative disorders, including Alzheimer’s disease (AD), PD (Jucker and Walker 2013), and MSA (Prusiner et al. 2015; Woerman et al. 2018). These disorders share key molecular mechanisms with prion diseases, particularly the abnormal misfolding and aggregation of specific proteins – such as amyloid-β and tau in AD, and aSyn in PD and MSA. Like prions, these misfolded proteins can form amyloid fibrils with β-sheet-rich structures, propagate in a prion-like manner, and induce pathology in neighboring cells (Jucker and Walker 2013). This commonality has led to the concept of “prion-like mechanisms” in non-TSE neurodegenerative diseases, expanding our understanding of how protein misfolding contributes to the onset and progression of these conditions.

The revolutionary concept of prions was not only that a protein could exist in at least two metastable conformations, conferring distinct phenotypes, but also that a specific protein conformation could induce the conversion of an alternate conformation into its state (i.e., permissive templating), thereby altering the phenotype. This is also the basis for the concept of ‘functional amyloids’, a concept put forward to capture the idea that the amyloid conformation of certain proteins may constitute ingenious ways biology evolved to expand protein functionality and that amyloids are not only associated with pathological conditions (Otzen and Riek 2019; Pena-Diaz et al. 2024). However, the protein conversion principle also underlies the infectious nature of prion diseases, where contamination with a misfolded protein initiates a cascade of conformational changes.

Today, the aggregation process is understood at a deeper level, particularly through the lens of phase separation and phase transition, whereby proteins demix from their surrounding liquid environment. At later stages, this process can progress to the formation of solid, aggregated states of proteins that can be considered *quasi*-irreversible (Alberti and Hyman 2021; Patel et al. 2015). Such structural transitions can lead to a loss of normal function and, in some cases, also to the gain of toxic function. Both outcomes (loss and gain of function) are implicated as likely in pathologies such as cancer, diabetes, infections, and neurodegenerative disorders, including PD. These insights not only redefine our understanding of the functional role of protein folding but also pave the way for therapeutic strategies that aim to modulate conformational transitions.

### Proteostasis: protein folding and unfolding in the crowded cellular environment

The process of protein aggregation has been extensively studied in vitro over the years. It has been known for some time that the assembly process of protein aggregates follows a sigmoidal curve, with an initial lag phase, rapid exponential growth, and eventual plateau as monomeric proteins are depleted (Serio et al. 2000; Conway et al. 2000). However, the mechanisms governing protein folding and unfolding in the crowded cellular environment — where concentrations of proteins and other biomolecules are extremely high—remain less understood.

Nonetheless, evidence suggests that similar kinetics may underlie the molecular and symptomatic progression observed for neurodegenerative diseases. Importantly, molecular alterations likely occur well before the onset of clinically apparent symptoms within a lag phase corresponding to a preclinical disease stage (Kalia and Lang 2015). This lag phase may represent a critical therapeutic window and intervening at this stage may prevent the exponential amplification of aggregated species, some of which are thought to be associated with cellular damage.

Cells must tightly regulate protein production, folding, clearance, and disaggregation in order to maintain protein homeostasis or proteostasis (Balch et al. 2008). This includes ensuring proper folding in diverse subcellular compartments and detecting and addressing instances of protein misfolding. The cellular proteostasis network encompasses molecular chaperones, degradation pathways such as the ubiquitin-proteasome system, and autophagy (Gidalevitz et al. 2010). This network also integrates signaling pathways that allow cells to respond to environmental perturbations that disrupt proteostasis, thereby mitigating damage and preventing cellular toxicity.

By targeting the molecular triggers of protein misfolding, it may be possible to halt the progression of aggregation at its earliest stages. Therefore, developing biomarkers capable of detecting these preclinical molecular changes would also enable early intervention, providing a promising avenue for the discovery of disease-modifying therapies (DMTs).

### Protein aggregation: a hallmark of neurodegenerative diseases

**Fig. 1:**
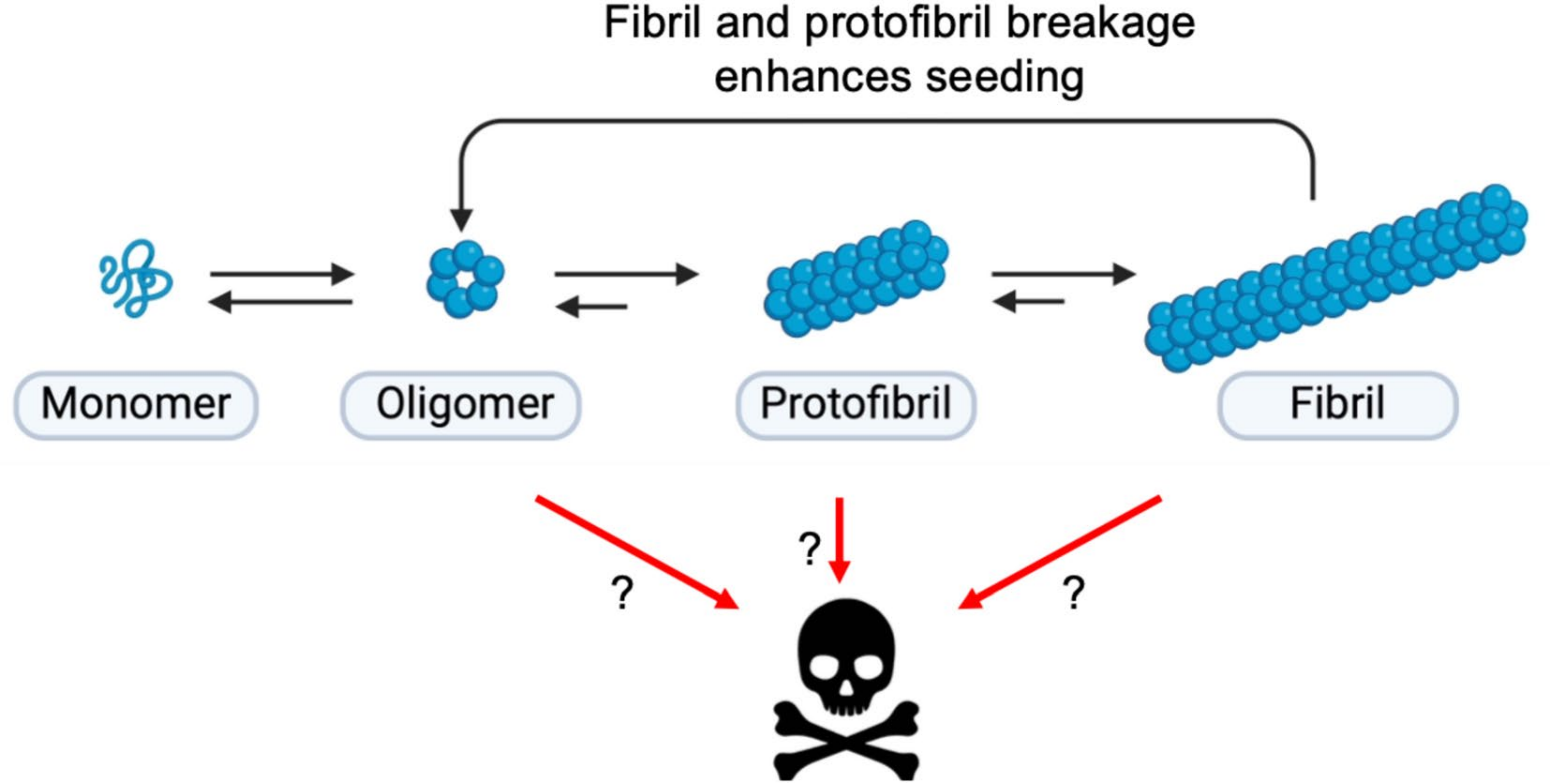
Illustration of protein aggregation and toxicity mechanisms. Proteins, in their monomeric state, can undergo conformational changes that trigger intermolecular interactions. In one possible pathway leading to self-assembly, proteins may form oligomeric species which, subsequently, assemble into protofibrils and eventually into typical amyloid fibrils, the most stable form. Amyloid fibrils may break, releasing oligomeric species that may contribute for feeding the cycle, thereby seeding further aggregation. During this process, it is thought that certain aggregated species may exhibit cytotoxic properties, but it is still unclear what are the most toxic species

Under various conditions, including environmental stressors, genetic mutations, post-translational modifications, or simply the aging process, the protein homeostasis (proteostasis) network may fail. This can lead to protein misfolding and the adoption of aberrant conformations, initiating aggregation processes. Some of the resulting aggregated species are thought to be cytotoxic (Fig. 1), leading to neuronal dysfunction and death in neurodegenerative diseases.

Protein aggregation is a common feature in various neurodegenerative diseases, including Alzheimer’s disease (AD), PD, and rarer conditions such as prion diseases, all of which have devastating consequences for the brain. A shared hallmark of neurodegenerative diseases, beyond region-specific neuronal death, is the accumulation of protein aggregates in the brain, and in some cases, even in peripheral tissues. However, it is now widely accepted that protein pathology in the various neurodegenerative diseases is more complex than initially thought and that multiple pathologies/co-pathologies can occur (Spires-Jones et al. 2017), especially with aging (Table 1). This understanding has strong implications for diagnosis and may prove useful also for disease classification (please see below).

**Table 1:** Major neurodegenerative diseases of the brain and associated proteinopathies. Diseases, affected brain areas, and pathological hallmarks (typical and as co-pathologies) are indicated. The percentage of co-pathologies vary depending on the study, so we did not include this information (adapted from (Robinson et al. 2018, 2023; spires-Jones et al. 2017)).

Neurodegenerative disease	Major brain regions affected	Typical protein pathology (and location)	Common co-pathologies
AD	Neocortex, allocortex, basal ganglia, dienchephalon, brain stem, and cerebellum Transentorhinal cortex, hippocampus, neocortex	Ab plaques (extracellular) and cerebral angiopathy (basement membrane of blood vessels) Tau neurofibrillary tangles (intracellular, mostly in neurons)	aSyn TDP-43
Tauopathies (e.g. FTLD-tau, Pick’s disease, PSP, FTD-17)	Frontal and temporal cortex, basal ganglia, and brain stem	Tau (cytoplasmic, in neurons and glia)	Ab aSyn TDP-43
Synucleinopathies	PD - brain stem, substantia nigra, cortex DLB - cortex and lower brain regions	aSyn – Lewy pathology (cell body, neurites, and nuclear, in neurons and glial cells)	Ab Tau TDP-43
	MSA-P - mainly in the striatum and substantia nigra; gray and white matter pathology MSA-C - mainly in the pons, medulla oblongata, and the cerebellum; gray and white matter pathology	aSyn glial cytoplasmic inclusions (GCIs). Papp-Lantos bodies. Some neuronal aSyn pathology (also nuclear).	Ab Tau TDP-43
HD	Caudate nucleus, putamen, globus pallidus, cerebellum, brain stem	huntingtin inclusions (intranuclear and cytosolic)	Tau aSyn
ALS	Motor cortex and other cortical regions, spinal cord	TDP-43, FUS, SOD1, ubiquilin, C9orf72 (cytoplasmic and nuclear inclusions in neurons and glia)	Tau aSyn
Prion diseases	Cortex, striatum, cerebellum, and spinal chord	PrP^Sc^ aggregates Spongiform changes	Tau Ab

Cross-seeding phenomena, where misfolded species of one protein can template and induce the aggregation of another protein, have been increasingly recognized as a potential contributor to co-pathologies observed in aging and neurodegenerative diseases. For instance, α-synuclein and tau aggregates have been shown to interact and cross-seed each other, potentially amplifying the complexity of proteinopathies (Moussaud et al. 2014; Williams et al. 2020). This interconnection may explain why patients often present with overlapping pathologies, such as tauopathy and synucleinopathy in advanced AD or Lewy body dementia (Bellomo et al. 2024; Irwin and Hurtig 2018; Spires-Jones et al. 2017; Anastassiadis et al. 2024; Maldonado-Diaz et al. 2024; Robinson et al. 2023). Understanding cross-seeding mechanisms is critical for elucidating the molecular underpinnings of disease heterogeneity and developing targeted therapeutic strategies.

In PD, dementia with Lewy bodies (DLB), pure autonomic failure (PAF) and multiple system atrophy (MSA), collectively referred to as synucleinopathies, α-synuclein (aSyn) is the key aggregating protein. Its misfolding and subsequent aggregation into oligomeric and fibrillar species represent central pathogenic events. These aggregates not only disrupt cellular functions but also contribute to the progressive neuronal loss observed in this disorder. Understanding how aSyn misfolds, aggregates, and interacts with the proteostasis network is therefore critical for developing effective therapeutic strategies to combat PD and related synucleinopathies.

The recognition that misfolded proteins can self-propagate through templated conversion has revolutionized not only our understanding of protein aggregation but also its potential diagnostic applications. Building on this knowledge, the development of seeding amplification assays (SAA) has emerged as a powerful tool for detecting the presence of misfolded and aggregated proteins associated with neurodegenerative diseases (Frey et al. 2023; Huang et al. 2024; Vascellari et al. 2022). These assays exploit the ability of pathological protein aggregates to induce the misfolding of their soluble monomeric counterparts, amplifying even minute amounts of disease-related species for detection.

SAAs, such as Real-Time Quaking-Induced Conversion (RT-QuIC) and Protein Misfolding Cyclic Amplification (PMCA), have demonstrated remarkable sensitivity and specificity in identifying disease-associated aggregates in various biological samples, including cerebrospinal fluid, blood, and even skin biopsies (Concha-Marambio et al. 2023; Frey et al. 2023; Huang et al. 2024; Vivacqua et al. 2023). Recent studies further highlight the potential of these techniques to advance the understanding of disease heterogeneity and identify reliable biomarkers for early diagnosis (Coysh and Mead 2022; Mok et al. 2023). In addition, using SAAs to study comorbidities (Anastassiadis et al. 2024) provides valuable insights into the synergistic propagation of different protein aggregates, which may help explain the overlapping phenotypes seen in patients.

For instance, a cross-sectional study assessing participants from the Parkinson’s Progression Markers Initiative (PPMI) cohort utilized aSyn seed amplification to reveal significant heterogeneity among individuals with PD. The findings underscored how SAAs may possibly distinguish between clinical subgroups, offering valuable insights into disease progression and variability (Siderowf et al. 2023).

Similarly, another study suggests that propagative aSyn seeds may be detected in serum samples, presenting a minimally invasive biomarker for synucleinopathies (Okuzumi et al. 2023). Although some of these assays still require more widespread validation, there is hope that SAAs may be used as a diagnostic tool capable of identifying disease-associated seeds in peripheral tissues, broadening their applicability in clinical settings.

These methods are paving the way for earlier and more accurate diagnoses, often before clinical symptoms manifest. However, it is important to acknowledge and study the fact that many asymptomatic individuals with evidence of protein aggregation in the brain (e.g., Ab or aSyn) may never evolve to develop neurodegeneration or even to a clinical disease state. Understanding the predictive and even protective factors that influence this conversion will be critical in the process of making accurate early diagnoses of neurodegenerative diseases. Early diagnosis is crucial for understanding disease progression and enabling the timely testing of emerging therapies. By identifying pathological changes at pre-symptomatic stages, these techniques hold the potential to transform the therapeutic landscape, facilitating interventions that target the molecular triggers of neurodegeneration before irreversible damage occurs. Ultimately, such advances bring us closer to developing more effective and personalized treatment strategies.

### Etiology of complex diseases and the absence of effective therapies

Although some neurodegenerative diseases have been described for over a century (and over two centuries in the case of PD), we still lack detailed insights into the molecular mechanisms that trigger these conditions. This knowledge gap has hindered the development of therapies capable of preventing or halting disease progression, despite thousands of clinical trials conducted to date. Only recently have faint signs of success emerged (Sims et al. 2023; van Dyck et al. 2023), underscoring the necessity of continuing to elucidate the molecular underpinnings of these disorders.

Genetic factors account for only a small fraction (~ 10%) of mendelian cases of PD or AD. While some conditions, such as Huntington’s disease, are purely genetic and follow Mendelian inheritance patterns, most neurodegenerative diseases are complex. In these conditions, a combination of genetic variations—each insufficient to cause disease alone—interacts with environmental factors to increase disease risk. Most neurodegenerative diseases, including PD, belong to this category.

The molecular processes associated with aging are the most significant risk factors for neurodegenerative diseases. Additionally, environmental factors can induce epigenetic changes that contribute to the onset of these disorders.

### From James Parkinson to the biological classification of PD

James Parkinson first described the disease in 1817 as “paralysis agitans”. However, the condition was formally named later by Jean-Martin Charcot, regarded as the “father of modern neurology,” who recognized the pivotal contribution of Parkinson’s work. Charcot refined the definition of the disease and identified bradykinesia as a critical feature.

Today, PD is understood as a highly complex disorder. While the motor symptoms are the hallmark leading to diagnosis, prodromal phases exhibit signs such as hyposmia, REM sleep disturbances, gastrointestinal and behavioral changes. These early symptoms implicate multiple brain regions and even peripheral organs, such as the gut, before involvement of the dopaminergic substantia nigra. This region of the brain experiences extensive neuronal loss in PD, with the resulting dopamine deficiency contributing to the motor deficits characteristic of the disease.

**Fig. 2:**
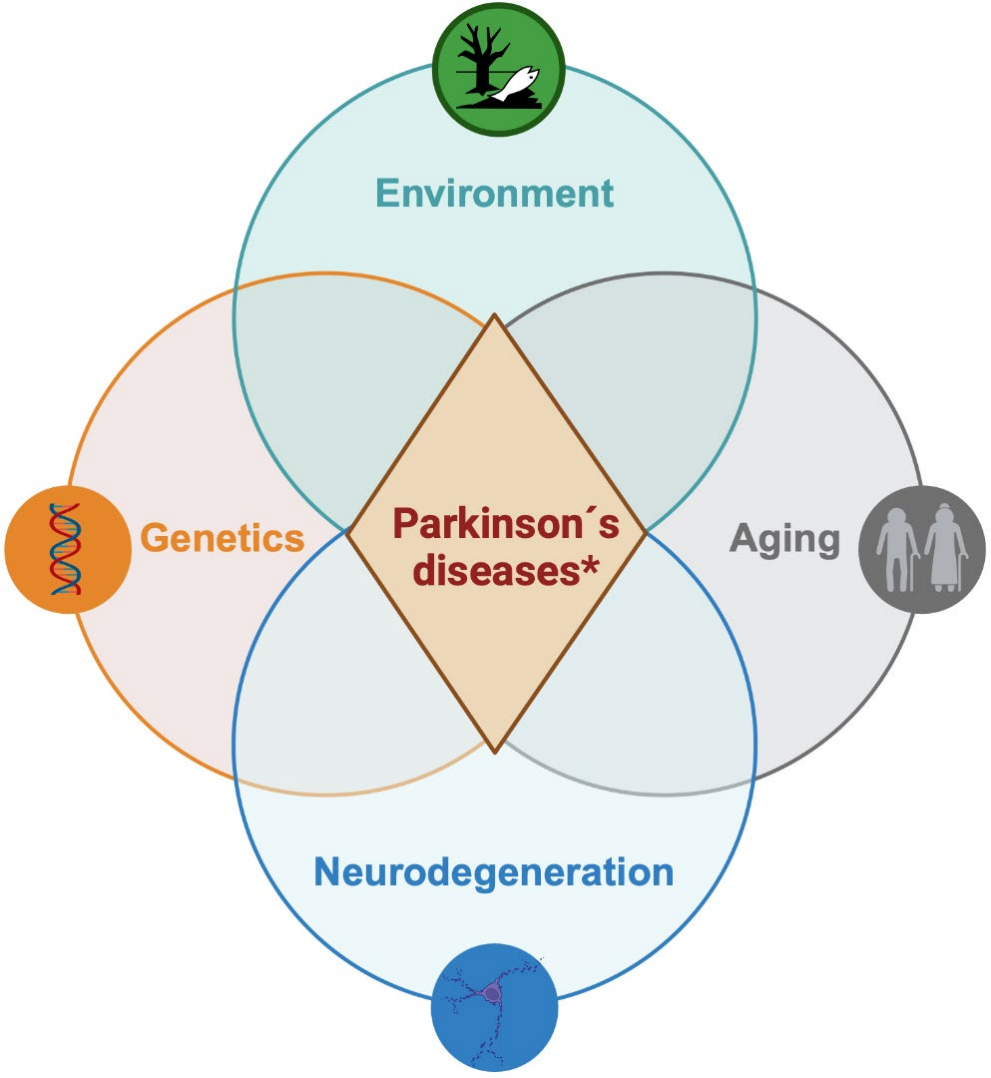
Factors contributing to the onset of PD. Aging, neurodegeneration, genetics, and environmental factors, contribute to the risk of developing particular forms of PD, suggesting the disease should be considered a heterogeneous group of related disorders (*Parkinson’s diseases) rather than a single disease entity

Advances in understanding of genetic factors, environmental factors, and factors contributing to neurodegeneration suggest that PD should not be considered a singular homogeneous condition but rather a complex multifaceted group of related disorders arising due to the combination of various complex components (i.e. ‘Parkinson’s diseases’) (Fig. 2). Importantly, various pathophysiological mechanisms have been associated with PD and they are likely preferentially associated with specific factors. Consequently, there is an increasing need for a broad set of criteria to classify and define PD based on biological markers rather than relying solely on descriptive, often subjective, clinical criteria.

In recent years, several groups of international leaders have proposed classification systems neurodegenerative diseases, beginning with AD (Jack et al. 2016, 2024) and most recently including PD. These PD-related proposals, initially designed for research purposes, are meant to serve as a foundation for diagnosis and differentiation between distinct PD subtypes (Hoglinger et al. 2024; Lang et al. 2024; Simuni et al. 2024). As new biomarkers become available, these classification systems will continue to evolve and it is likely, and desirable, they become integrated in routine clinical practice.

### Studying aSyn aggregation in cell models

Several molecular mechanisms have been implicated in the etiology of PD, including mitochondrial dysfunction, oxidative stress, neuroinflammation, and alterations in the function of pathways involved in proteostasis. The aggregation of aSyn into Lewy neurites and Lewy bodies (Lewy pathology), the protein aggregates characteristic of PD and dementia with Lewy bodies, is also considered a key mechanism in PD. More recently, a hypothesis was proposed suggesting that the progression of clinical symptoms is related to the progressive spread of Lewy pathology accumulation throughout the brain (Braak et al. 2003). This hypothesis has been widely adopted but has limitations, as a significant percentage of PD cases do not follow the pattern of pathology spread proposed. Moreover, we cannot conclusively state that Lewy pathology is responsible for neurodegeneration in PD and other synucleinopathies, as it may be simply a bystander, an epiphenomenon, or even a protective cellular response.

However, based on genetic evidence, where point mutations or duplications/triplications of the gene encoding aSyn are associated with PD (Xu et al. 2015), it is unavoidable to assume that this protein plays a central role in synucleinopathies. Since the association of the protein with PD in 1997 (Polymeropoulos et al. 1997; Spillantini et al. 1997), various laboratory models have been developed to study the biology of aSyn and the molecular mechanisms linking it to other disorders, such as DLB and MSA.

### Super-resolution microscopy to study the ‘architecture’ of aSyn aggregates

In 1912, long before the identification of aSyn as a major component of protein aggregates accumulating in the brains of PD patients, Friedrich Lewy observed and described these aggregates. Later, with the identification of aSyn in Lewy bodies, several laboratory models were developed to mimic the protein aggregation and enable its study.

Over the years, several simple cellular models were developed for studying aSyn aggregate formation, either directly, through fusion with fluorescent proteins, or indirectly, using immunocytochemistry techniques (Lazaro et al. 2014; Outeiro and Lindquist 2003; Outeiro et al. 2008). With these models, it has been possible to study the effects of mutations, post-translational modifications, and environmental factors on aSyn aggregation. However, it has become evident that larger aggregates may not necessarily be the most cytotoxic. Therefore, it is essential to use more sophisticated microscopy techniques, such as super-resolution microscopy, to identify and study smaller aggregates, investigate their molecular architecture, and identify factors that can interfere with their formation. In this regard, we have used techniques such as STED microscopy and, more recently, expansion microscopy, with innovative results (Lazaro et al. 2014, Weish et al. 2021).

### Posttranslational modifications: glycation as an example and potential therapeutic target in PD

Another growing area of research is the study of the effect of posttranslational modifications (PTMs) as conformational modulators that can enhance the aggregation pathway of various proteins. This topic is particularly relevant in the context of aSyn, as several PTMs have been identified, including phosphorylation of serine at position 129 (pS129), which is considered a marker of pathology in laboratory models.

Among others, we have studied a type of modification that may be particularly important in the context of PD, as it establishes a potential link with diabetes, a disease associated with an increased risk of developing PD (Cullinane et al. 2023). This PTM is glycation, which consists of a non-enzymatic reaction between sugars and specific amino acids (Vicente Miranda and Outeiro 2010). Glucose metabolism inherently generates methylglyoxal, a highly reactive aldehyde that modifies lysine or arginine residues. While aSyn does not have arginine residues in its primary sequence, it does possess 15 lysine residues. Through studies in various models, including yeast, human cell lines, iPS cell lines, *Drosophila*, and mice, we demonstrated that methylglyoxal glycation promotes the formation of aSyn oligomers and increases the protein’s toxicity (Farzadfard et al. 2022; Vicente Miranda et al. 2017). These studies suggest new potential therapeutic targets that need to be explored further.

Our results and those of many others in the field, suggest that glycation may occur as a consequence of changes in environmental factors (such as diabetes or other metabolic alterations), causing an imbalance in proteostasis, leading to aSyn oligomerization and its toxicity. Therefore, identifying molecules capable of interfering with the available amount of methylglyoxal, thereby reducing glycation, may constitute a valid strategy in the context of synucleinopathies (Farzadfard et al. 2022; Vicente Miranda et al. 2017).

### Conclusions and outlook

Over the years, the scientific community has made tremendous progress in our understanding of molecular mechanisms involved in neurodegenerative diseases. While laboratory models are essential for testing basic molecular mechanisms, it has also become evident that we need to use and develop more complex models that aim to recapitulate the genetic and cellular context of cells in the complex environment of the human brain. In turn, this requires the development of disease classification systems that capture the underlying biology/pathobiology so that we can attempt to recapitulate disease in model systems. Ultimately, the hope is that we can diagnose diseases early to maximize our chances of therapeutic success.

Combining “omics” technologies with advanced computational tools offers new avenues for understanding and treating neurodegenerative diseases. In the future, by analyzing molecular data specific to individual patients, these approaches can help identify early disease markers and enable the development of targeted therapeutic strategies. This personalized framework may also improve drug discovery, focusing on interventions that address the underlying mechanisms of protein misfolding and aggregation. As these tools evolve, they hold increasing potential for reshaping the classification and treatment of these complex disorders.
